# How Engagement Changes Over Time in a Digital Eating Disorder App: Observational Study

**DOI:** 10.2196/68824

**Published:** 2025-09-30

**Authors:** Rachael E Flatt, Laura M Thornton, Jenna Tregarthen, Stuart Argue, Cynthia M Bulik

**Affiliations:** 1 Department of Psychiatry, University of North Carolina at Chapel Hill, Chapel Hill, NC, United States; 2 Recovery Record (United States), Palo Alto, CA, United States; 3 Department of Medical Epidemiology and Biostatistics, Karolinska Institutet, Nobels väg 12A, Stockholm, 17177, Sweden, 46 9195935922; 4 Department of Nutrition, University of North Carolina at Chapel Hill, 101 Manning Drive, Chapel Hill, NC, 27599, United States, 1 984-974-3233

**Keywords:** eating disorders, wearable technology, mobile application, engagement, baseline

## Abstract

**Background:**

Engagement with digital mental health interventions is often measured as a summary-level variable and remains underresearched despite its importance for meaningful symptom change. This study deepens understanding of engagement in a digital eating disorder intervention, recovery record, by measuring engagement with unique components of the app, on 2 different devices (phone and watch), and at a summary level.

**Objective:**

This study described and modeled how individuals engaged with the app across a variety of measures of engagement and identified baseline predictors of engagement.

**Methods:**

Participants with current binge-eating behavior were recruited as part of the Binge Eating Genetics Initiative study to use a digital eating disorder intervention for 4 weeks. Demographic and severity of illness variables were captured in the baseline survey at enrollment, and engagement data were captured through both an iPhone and Apple Watch version of the intervention. Engagement was characterized by log type (urge, behavior, mood, or meal), device type (logs on phone or watch), and overall usage (total logs) and averaged each week for 4 weeks. Descriptives were tabulated for demographic and engagement variables, and multilevel growth models were conducted for each measure of engagement with baseline characteristics and time as predictors.

**Results:**

Participants (N=893) self-reported as primarily White (743/871, 85%), non-Hispanic (801/893, 90%), females (772/893, 87%) with a mean age of 29.6 (SD 7.4) years and mean current BMI of 32.5 (SD 9.8) kg/m^2^ and used the app for a mean of 24 days. Most logs were captured on phones (217,143/225,927; 96%), and mood logs were the most used app component (174,818/282,136; 62% of logs). All measures of engagement declined over time, as illustrated by the visualizations, but each measure of engagement illustrated unique participant trajectories over time. Time was a significant negative predictor in every multilevel model. Sex and ethnicity were also significant predictors across several measures of engagement, with female and Hispanic participants demonstrating greater engagement than male and non-Hispanic counterparts. Other baseline characteristics (age, current BMI, and binge episodes in the past 28 days) were significant predictors of 1 measure of engagement each.

**Conclusions:**

This study highlighted that engagement is far more complex and nuanced than is typically described in research, and that specific components and mode of delivery may have unique engagement profiles and predictors. Future work would benefit from developing early engagement models informed by baseline characteristics to predict intervention outcomes, thereby tailoring digital eating disorder interventions at the individual level.

## Introduction

### Background

Digital interventions hold substantial promise for addressing mental health disorders, including eating disorders (EDs). Traditional face-to-face ED treatment is often cost-prohibitive, especially for the uninsured, difficult to access, not scalable, retrospective in nature, and unable to offer support in real time when patients need it most [1-3]. Digital ED interventions address these obstacles by offering evidence-based and in-the-moment treatment options that are affordable and accessible [4-7]. A crucial aspect of digital interventions is engagement [8,9], which parallels measures of retention and adherence in face-to-face treatments. Just like individuals attend sessions, complete self-monitoring forms for home practice, and apply learned skills outside of sessions, digital interventions for EDs can measure similar and expanded forms of engagement that may be key to achieving and maintaining treatment gains.

Similar to observations in the broader digital mental health tool literature, engagement in digital ED interventions is low [6], the definitions used to describe engagement are heterogeneous and mostly limited to static or summary-level variables, and predictors of engagement are not well researched. Thus, this study analyzes engagement in a digital ED intervention, recovery record, over the course of a month for individuals with binge-type EDs. Specifically, we model longitudinal growth curves to examine the dynamic trajectories of several measures of engagement and subsequently identify baseline predictors.

### Binge-Type Eating Disorders

Bulimia nervosa (BN) and binge eating disorder (BED) affect between 1.0%‐3.5% of the population [10,11], disproportionately affect females, and have a typical age of onset in the late teens and early 20s [12]. If left untreated, both disorders carry significant psychological and medical implications, high health care use, and high relapse rates [13,14]. Given that the courses of these disorders are dynamic in nature (eg, diagnostic shift and changes in temporal patterns of binge eating and compensatory behaviors), digital ED interventions need to be flexible such that individuals can engage with them when they need them most in the moment. Digital ED interventions can help address critical gaps in accessing care in a timely fashion; however, it is essential that we begin to refine our understanding and conceptualizations of how individuals with EDs engage with these tools to support future work that facilitates uptake, usability, acceptability, and positive clinical outcomes.

### Engagement in Digital Eating Disorder Interventions

Engagement, broadly defined in this paper as the ways and extent to which an individual uses a digital intervention, is frequently cited as a key challenge in digital mental health tools [8,15,16]. Although different terms are used to describe how an individual uses a digital mental health app (engagement, app usage, adherence, etc), the term engagement is used throughout this paper for consistency. Across studies reporting on engagement in digital ED interventions, barriers to initiating engagement identified across qualitative and quantitative studies of digital ED interventions mirrored those of other digital mental health interventions and included logistical constraints such as cost, accessibility, usability or functionality [17], time [18], and privacy of personal health information [19]. Perceived barriers include treatment credibility and expectancy [20], motivation, accountability, content and feature preferences [17], severity of illness, trust of the intervention [18], satisfaction, intervention personalization, and ease of use [19]. Once individuals initiate engagement with an app, several papers also cite maintaining engagement as a significant challenge: the median percentage of users in a review of treatment studies of digital ED interventions where all prescribed modules or activities were completed was only 36% [6]. Greater engagement is shown to be associated with better treatment outcomes [21,22], suggesting that maintaining engagement is important for users seeking meaningful change in their ED symptoms. Notably, most of these definitions only captured summary-level or endpoint measurements of engagement, so little is known about how engagement changes over time and if changes in engagement contribute to treatment gains.

Researchers also contend with methodological challenges, namely that there are seemingly endless conceptualizations and both qualitative and quantitative measurements of engagement [16,23,24], and most studies evaluating engagement do not capture multiple data streams to illustrate dynamics. The types of engagement data collected also vary widely depending on the features and content delivered to users through the intervention, the data users consent to provide, and the mode of delivery (eg, through a smartphone app, wearable technology, and web-based browser). In the context of digital ED interventions, the definitions of engagement vary widely: for example, studies defined engagement as number of modules completed [21]; total number of logs completed, total number of days active with the app, and length of time using the app [22]; interactivity and usability [25]; and total app views and total number of meal logs [26]; among others. Relatedly, apps and devices can limit or bias the engagement data that are reported due to differences in data capture. As an example, apps may capture the number of logins without adequately capturing a logout time, thus biasing the data toward extended periods of time where it appears that users are logged on and engaged. In research studies, investigative teams also typically use strategies such as reminders, phone calls, and compensation to boost user engagement [6], which may inflate overall engagement results compared with naturalistic app use. As a result, the field has yet to elucidate common themes about temporal changes in engagement beyond summary-level variables.

### Recovery Record Engagement Research

This study uses data collected through recovery record [3], a widely used evidence-based ED app; however, only 2 studies assessed participant engagement in recovery record to date. The first qualitative study explored engagement in a Danish-translated version of Recovery Record in participants with anorexia nervosa (AN) or BN [27], the majority of whom used the app between 1 and 4 months. Participants reported that engaging with the app helped them confront the ED or log meals more constructively, leading to less concern with caloric intake, but also reported that engagement could be obstructive by increasing obsessions with logging or by giving participants ideas about other compensatory behaviors (eg, participant sees “excessive exercise” as an option to log and begins to feel an urge to exercise). The second study [22] evaluated participants’ engagement with recovery record in a sample of recovery record users as part of a larger randomized controlled trial testing efficacy of the app. The total number of meal logs and total number of days the app was used significantly and positively mediated the treatment effect on clinical response 8 weeks later, indicating that this greater engagement could lead to more positive treatment outcomes. However, only 3 measurements of engagement were sufficiently tested as mediators, and the study did not describe or evaluate changes in engagement over time.

Despite the multitude of interactive features that most digital ED apps offer, including recovery record, most digital ED research has focused on heterogenous summary-level measurements of engagement (eg, number of days the app was used), and the analyses do not reflect the dynamic nature of engagement. In addition, baseline contextual factors that may influence engagement trajectories have not yet been thoroughly explored with digital ED interventions. As a result, valuable information pertaining to engagement trajectories is mostly unavailable, and we may fail to capture how different types of engagement change over time and key participant characteristics that are associated with engagement. This study addresses these issues by defining several types of engagement based on the available interactive features in recovery record, modeling trajectories for each measure of engagement, and identifying key baseline predictors of engagement.

### Aims

The aims of this study were to: (1) describe characteristics of the sample and how individuals engaged with the app across a variety of measures of engagement, (2) model the trajectories of engagement over the 30-day course of the study, and (3) identify baseline demographic and ED symptom predictors of engagement. Based on previously published literature, we hypothesized that individuals will generally demonstrate downward trajectories of engagement across the study period. However, the dearth of evidence on engagement in digital ED interventions and in recovery record does not support more specific a priori hypotheses for baseline predictors of engagement.

## Methods

### Participants

Participants were recruited as part of a larger parent case-only trial, the Binge Eating Genetics Initiative (BEGIN) study. The full study protocol for the parent trial is available elsewhere [28]. Briefly, the BEGIN study sought to integrate genetic, microbiome, phenotypic, and behavioral data for individuals with binge-type EDs. Pertinent to this paper, participants used recovery record through an iPhone and Apple Watch to collect actively logged behavioral and affective data in addition to passive data on heart rate and steps as foundations for just-in-time adaptive interventions (refer to Bulik et al [28] and Flatt et al [29] for more details on the technology component of the study as well as photos of the app on the Apple Watch). Inclusion criteria for this study included (1) current binge eating, (2) lifetime diagnosis of either BN or binge-eating disorder, (3) US resident, (4) between 18 and 45 years old, (5) reads and speaks English, (6) current iPhone user, (7) ambulatory, and (8) willing and able to participate in the study, wear an Apple Watch, and use recovery record. One additional criterion for analysis in this study was completion of at least 1 log on Recovery Record.

Exclusion criteria included (1) currently pregnant or breastfeeding, (2) history of bariatric surgery, (3) current use of hormone therapy, (4) inpatient treatment or hospitalization for ED in the 2 weeks before enrollment, (5) current suicidality, and (6) antibiotic or probiotic use at enrollment. Of note, some exclusion criteria were related to other aspects of the parent trial (eg, microbiome testing).

### Procedures

Participants were recruited primarily through recovery record, social media posts on Facebook and X (formerly Twitter), and emails through a University of North Carolina participant registry listserve. After completing three logs on the recovery record and demonstrating initial engagement with the app, individuals were sent a message from the app with a brief description of the study and were invited to complete online consent forms followed by a screener for lifetime ED diagnosis using the ED100Kv2 [30,31]. Those who met all inclusion criteria were offered a second consent and the option to participate in the study. They were subsequently asked to complete a baseline questionnaire consisting of several demographic questions and measures assessing ED and general psychopathology (depression, anxiety, and attention-deficit or hyperactivity disorder screeners). Packages containing an Apple Watch (if they did not already have one) and sampling kits (for genetic and microbiome sampling) were then sent to participants within the first few days of enrollment and included a set of instructions to set up and configure recovery record on their Apple Watch.

Participants were asked to use a recovery record, a cognitive behavioral therapy-based application designed to support individuals with an ED, through an iPhone and an Apple Watch (first generation) with a version of the recovery record app designed specifically for the parent trial. Participants were asked to log ED urges and behaviors including binge eating and compensatory behaviors (vomiting, diuretic and laxative misuse, excessive exercise, and fasting) and their mood through the recovery record app on the Apple Watch, although these logs could also be completed on the iPhone app. In addition, participants logged their meals on the iPhone app rather than the Apple Watch given the larger screen. Skills (eg, distraction, mindful breathing, emotion regulation, and challenging negative thoughts) were also available for participants to use. Individuals who were already working with a clinician outside of the study could connect with them through the recovery record app; however, this was not included as a part of engagement data collection. If participants had technical difficulties with the app, they were instructed to reach out to the study team. Participants also had the ability to personalize the app and enable push notifications or daily reminders; however, they were not required to enable these notifications as part of the study. All app content was accessible to participants throughout the duration of the study. Midpoint and endpoint questionnaires assessing ED, mood, and anxiety symptoms were administered 15 and 30 days, respectively, after enrollment.

### Measures

Demographic information was collected via a questionnaire administered at baseline on age, gender, race, and ethnicity. Biological sex at birth was determined via saliva sample for the genetic testing component of the parent study [32]. Current BMI was calculated at baseline with self-reported height and weight. Lifetime ED diagnosis was determined by algorithm using items from the ED100Kv2 [30,31].

Information on current ED symptomatology was collected through the Eating Disorders Examination Questionnaire (EDE-Q) [33] administered in the baseline, midpoint, and endpoint questionnaires. The EDE-Q is a widely used self-report ED questionnaire and has demonstrated good validity and reliability in community samples [34]. In total, 28 items cover various aspects of ED pathology including weight, shape, and eating concerns, current BMI, and ED behaviors including binge eating and compensatory behavior frequency (vomiting, fasting, excessive exercise, diuretic, and laxative misuse) over the past 28 days. The EDE-Q global score [35,36] was calculated from the 22 Likert-scale items (0=no days, 6=every day for items assessing frequency OR 0=not at all, 6=markedly for items assessing distress or impairment).

### Engagement

To extend the focus of existing literature on summary-level variables and broaden the types of engagement measured, we assessed the interactive features Recovery Record offers. In addition, since a central component of evidence-based treatment for binge-type EDs is self-monitoring of meals, mood, urges, and ED behaviors to help patients identify triggers and maintaining factors of the ED [33], participants were explicitly instructed to focus on logging these aspects through the app. All data used to describe types of engagement were collected through the recovery record app on both the iPhone and the Apple Watch. As such, the types and definitions of engagement used for this study, presented in Table 1, focus on use of meal, mood, and behavioral logs, overall usage, and through which mode of delivery (ie, iPhone or Apple Watch). All measures of engagement defined in Table 1 take an approach of mean usage (ie, the average number of times per day in 1 week that an individual used part of the app) to help capture change in engagement over time. To characterize how participants engaged with the app, each type of engagement was tabulated over week-long periods, thus participants can have up to 4 repeated measures of engagement for weeks 1-4. However, since participants received their Apple Watch devices approximately 1 week into the study, we only include data from weeks 2‐4; therefore, participants have up to 3 repeated measures of engagement in the watch log models only. Week 1 data collection began the day after enrollment.

**Table 1.** Engagement terms and descriptions of engagement definitions, each used as the dependent variable in separate multilevel models.

Engagement measure	Definition of engagement
Log type	
Mean urge logs	Number of times a participant logged an urge during a day, averaged over a 7-day timespan
Mean eating behavior logs	Number of times a participant logged an eating behavior during a day, averaged over a 7-day timespan
Mean mood logs	Number of times a participant logged a mood during a day, averaged over a 7-day timespan
Mean meal logs	Number of times a participant logged a meal during a day, averaged over a 7-day timespan
Device type	
Mean phone logs	Number of times the app was used on the iPhone in any capacity during a day, averaged over a 7-day timespan
Mean watch logs^a^	Number of times the app was used on the Apple Watch in any capacity during a day, averaged over a 7-day timespan
Mean use	The number of times the app was used in any capacity on the iPhone or Watch during a day, averaged over a 7-day timespan

a Due to participants receiving their Apple Watch devices ~1 week into the study, we only report data from weeks 2‐4.

### Data Analysis

All data preparation and analyses were conducted using SAS 9.4 (SAS Institute Inc) [37]. To prepare data for analysis, we first screened for unrealistic values of binge eating and compensatory behavior episodes reported at baseline (ie, 500 episodes reported in the past 28 days), and winsorized those values. For model estimation and interpretation purposes and to protect the privacy of participants with demographic characteristics with cell sizes <5, we included male and female participants who reported data for all baseline predictor variables (age, sex, ethnicity, current BMI, and baseline binge-eating episodes). For the engagement data, we screened for and removed duplicates and impossible or improbable measures of engagement (ie, future-dated timepoints, 1000 logs of the same event in 1 day); no imputation methods were used, since lack of engagement data at a given time point was not necessarily indicative of missingness.

To address aim 1, we first characterized the sample at baseline. Descriptive statistics (n’s, percentages, means, and SDs as appropriate depending on variable type) were provided on demographic variables (age, gender, sex, race, ethnicity, and current BMI), ED diagnosis, baseline ED psychopathology (EDE-Q global scores and number of binge-eating and compensatory behavior episodes in the past 28 days as measured by the EDE-Q), and for each measure of engagement listed in Table 1. We also performed a Poisson regression of the total number of days participants used recovery record in any capacity. Baseline predictors (age, sex, ethnicity, current BMI, and number of binge-eating episodes at baseline) were used as independent variables. Race was not used due to small cell sizes.

For aim 2, we analyzed the engagement data using multilevel growth models due to the nested structure of the data (up to 4 repeated measures within individuals for all measures of engagement except for mean watch logs, which is up to 3 repeated measures of engagement due to participants receiving their devices by the end of week 1), using the types of engagement included in Table 1 as the dependent variables. First, we visualized the data by plotting each measure of engagement over time (measured in weeks) using spaghetti plots to identify what type of functional form should be used (ie, linear and piecewise) for each model. For each measure of engagement, we began with unconditional multilevel models using the multilevel model PROC MIXED function. If the spaghetti plots were unclear as to what functional form should be used, we compared the intraclass correlation (ICC), Akaike Information Criterion (AIC), and Bayesian Information Criterion (BIC) values in the initial unconditional models to determine which functional form was most appropriate. Findings from the spaghetti plots and unconditional models were qualitatively summarized in the text to highlight relevant themes.

For aim 3, we expanded analyses to conditional models of engagement by including time (level 1 predictor measured in weeks) and the following baseline demographic predictors as time-invariant covariates (level 2 predictors): age, sex, ethnicity, and current BMI. Given that the parent sample recruited individuals with current binge eating, we then added baseline number of binge-eating episodes in the past 28 days from the EDE-Q as a measure of severity of illness to use as a level 2 time-invariant predictor of engagement. We mean centered age, current BMI, and baseline binge-eating episodes for ease of interpretation. For the categorical predictors, the reference variables were set to week 1 for time, participants who were categorized as male based on genotype for sex, and participants who self-identified as Hispanic for ethnicity. Predictors with significant fixed effects estimates were reported for each model, and themes are summarized in the text.

All multilevel models were fit using restricted maximum likelihood, the α level for fixed effects was set to 0.05, and we used Satterthwaite degrees of freedom approximations to reduce type 1 error rates [38]. We limited the models to fixed effects to help with estimation that was consistent across models, especially given that the predictors of interest were primarily level 2 time-invariant predictors within the context of this study and plan to further explore random effects in future analyses.

### Ethical Considerations

All study procedures were approved by the University of North Carolina Biomedical institutional review board (IRB# 17-0242 and 20-3229). All research activities were conducted in compliance with ethical standards, ensuring the privacy and confidentiality of participants’ data; all data were deidentified prior to analysis. Participants provided informed consent before initiating the study. They did not receive compensation for their participation, however, those who were sent an Apple Watch were able to keep the devices at the end of the study.

## Results

### Demographics and Sample Description

A total of 893 participants engaged with recovery record at least once during the study, had complete data for all baseline predictors, and were subsequently included in analyses. Participants in this study represented approximately three-fourths of the full sample recruited for the parent BEGIN study (n=1166). Of the 893 participants, 772 (86.5%) were assigned as female based on genotype and 121 (13.5%) as male; 680 (84.1%) self-identified as women, 120 (14.8%) as men, and 9 (1.1%) as nonbinary or third gender (n=84 did not report gender). The sample mostly identified as White (n=743/871, 85.3%), followed by more than 1 race (54/871, 6.2%), African American (35/871, 4.0%), Asian (33/871, 3.7%), and Native American or American Indian (6/871, 0.7%); and 22 did not report race. In addition, 92 of the 893 (10.3%) participants identified as Hispanic. The mean age of the sample was 29.6 (SD 7.4) years, and the mean current BMI was 32.5 (SD 9.8) kg/m^2^.

Across eating disorder characteristics, 78.3% (699/893) met ED100Kv2 criteria for lifetime BED, 26.9% (240/893) for lifetime BN, and 18.7% (167/893) for lifetime AN (participants could meet criteria for more than 1 ED diagnosis). The mean EDE-Q global score was 3.93 (SD 1.01). The mean EDE-Q subscale scores were 3.65 (SD 1.25) for eating concern, 2.90 (SD 1.58) for restraint, 4.71 (SD 1.12) for shape concern, and 4.44 (SD 1.12) for weight concern. At baseline, participants reported a mean of 12.92 (SD 9.57) binge episodes, 3.00 (SD 9.46) vomiting episodes, 0.89 (SD 0.43) laxative or diuretic misuse episodes, and 4.16 (SD 6.71) compulsive exercise episodes in the past 28 days as captured by the EDE-Q.

For measures of engagement, the sample used the recovery record app for an average of 24.09 (SD 7.18) days out of 30, with (209/893, 23.4%) participants using the app all 30 days. Across all participants included in this study, a total of 225,927 recovery record logs were captured over 4 weeks. Refer to Table 2 for descriptive data on the total sum of logs for each engagement variable over 4 weeks as well as the mean number of logs per day for each engagement variable over 4 weeks and by week. Notably, the majority of logs were completed on the iPhone (n=217,143/225,927 logs, 96.1%) rather than on the Apple Watch. After centering age, current BMI, and number of binge episodes at baseline, age (ß=.00, *χ*^2^_1_=5.74, false discovery rate [FDR]–adjusted *P*=.03) and sex (ß=.06, *χ*^2^_1_=8.18, FDR-adjusted *P*<.01) were significant predictors of the total number of days participants used the recovery record app. Specifically, participants who were older and female used the app a greater number of days.

**Table 2.** Total sum, median, and mean number of logs per day across 4 weeks and by week for each engagement variable.

Engagement variable	Total sum of logs over all 4 weeks, n	Logs per day over all 4 weeks, mean (SD)	Logs per day overall 4 weeks, median (IQR)	Logs per day during week 1 (n=893), mean (SD)	Logs per day during week 2 (n*=*835), mean (SD)	Logs per day during week 3 (n*=*784), mean (SD)	Logs per day during week 4 (n*=*681), mean (SD)	Percent change from week 1 to week 4, %
Log type								
Urge logs	14,855	0.7 (1.1)	0.3 (0.0-0.9)	0.8 (1.8)	0.8 (1.2)	0.6 (1.1)	0.4 (1.0)	−47.5
Behavior logs	25,420	1.1 (1.3)	0.7 (0.3-1.6)	1.5 (1.4)	1.3 (1.4)	0.1 (1.2)	0.7 (1.0)	−50.0
Mood logs	174,818	7.8 (6.4)	6.0 (2.9-9.6)	9.8 (6.5)	8.3 (6.5)	7.0 (6.1)	5.7 (5.5)	−41.6
Meal logs	67,043	3.0 (1.8)	3.0 (1.4-4.6)	3.6 (1.6)	3.1 (1.8)	2.8 (1.8)	2.4 (1.8)	−34.4
Device type								
Phone logs	217,143	9.7 (8.2)	8.0 (3.7-13.1)	12.4 (8.6)	10.2 (8.3)	8.5 (7.6)	7.0 (6.9)	−43.7
Watch logs^a^	6977	0.4 (0.9)	0.0 (0.0-0.4)	—^b^	0.7 (1.1)	0.4 (0.8)	0.2 (0.6)	−67.7
All logs	225,927	10.1 (8.4)	8.3 (3.9-13.7)	12.7 (8.8)	10.9 (8.6)	8.9 (7.8)	7.2 (7.1)	−43.3

a Due to participants receiving their Apple Watch devices ~1 week into the study, we only report watch log data from weeks 2‐4.

b Not available.

### Characterization of Engagement

To illustrate participant engagement over the course of 4 weeks (3 weeks for the watch logs) and to evaluate the functional forms to be used for subsequent conditional multilevel models, spaghetti plots for each measure of engagement were created using the mean number of logs over each week, seen in Multimedia Appendix 1. Across each measure, there was a general downward and linear trajectory of engagement across time. The mean meal logs plot had the most variability in individual trajectories and was initially difficult to discern a clear functional form. However, after visualizing the data via spaghetti plots in smaller groups of participants (ie, n=100) in combination with the data from Table 2, both linear and quadratic functional forms were tested in unconditional models. After comparing the ICC, AIC, and BIC, a linear functional form was determined to be the best fit for each measure of engagement and was used in the subsequent multilevel models detailed in the next section. Model fit statistics (ICC, AIC, and BIC) can be found for each model in Table S1 in Multimedia Appendix 2.

### Predictors of Engagement

Table 3 presents the results from the conditional models evaluating which baseline characteristics were significant predictors of engagement. The first set of models used demographics as predictors; the second set of models added an additional predictor, baseline binge episodes, which was used as a measure of illness severity. Age and current BMI regression coefficients and standard errors were calculated based on 5-year and 5 kg/m^2^ differences, respectively, to facilitate interpretation for more meaningful differences in engagement. However, baseline binge episodes were not altered given previous research that illustrates 1 binge episode per week results in a meaningful clinical difference in psychopathology for individuals with binge-type eating disorders [39].

Time, measured in weeks, was a significant negative predictor in nearly every instance, indicating that the number of weeks into the study consistently predicted a decline in engagement, regardless of how it was measured. Across demographic characteristics, sex and ethnicity were significant, positive predictors of mean mood, meal, and phone logs, as well as mean use. Mean-centered age was a significant positive predictor of mean meal log engagement such that those who were older logged more meals, and mean-centered current BMI was a significant negative predictor of mean urge logs such that those with higher BMIs at baseline logged fewer urges.

In the second set of models, the number of binge episodes at baseline was a significant, positive predictor of mean behavior logs. However, the number of binge episodes at baseline was not a significant predictor of any other measure of engagement, and there were no changes to significance for any other predictors when this variable was added to each engagement model. Notably, the model fit statistics and parameter estimates demonstrated negligible changes between models 1 and 2 for all measures of engagement.

**Table 3.** Multilevel model summaries for baseline demographic and severity of illness variables predicting engagement measures. The reference values for categorical variables were set to week 1, males, and individuals who identified as Hispanic. The watch log models only use data aggregated from weeks 2, 3, and 4 since most participants received their Apple Watch devices by the end of week 1. *P* values are presented as false discovery rate-corrected *P* values. Bolded values are significant.

Engagement variable	Week			Age^a^	Sex (female)	Ethnicity (Hispanic)	Current BMI^a^	Binge episodes
	2 b (95% CI)	3 b (95% CI)	4 b (95% CI)	b (95% CI)	b (95% CI)	b (95% CI)	b (95% CI)	b (95% CI)
Mean urge logs								
M1^b^	0.0 (−0.1 to 0.1)	−0.3^d^ (−0.3 to −0.2)	−0.5 (−0.5 to −0.4)	0.0 (0.0 to 0.0)	0.1 (−0.1 to 0.3)	0.0 (−0.2 to 0.2)	−0.1^d^ (−0.1 to 0.0)	—^e^
M2^c^	0.0 (−0.1 to 0.1)	−0.3^d^ (−0.3 to −0.2)	−0.5^d^ (−0.5 to −0.4)	0.0 (0.0 to 0.0)	0.1 (−0.1 to 0.3)	0.0 (−0.2 to 0.2)	−0.1^d^ (0.0 to 0.0)	0.0 (0.0 to 0.0)
Mean behavior logs								
M1	−0.3^d^ (−0.3 to −0.2)	−0.6^d^ (−0.7 to −0.5)	−0.9^d^ (−1.0 to −0.8)	0.0 (0.0 to 0.1)	0.0 (−0.2 to 0.2)	0.2 (−0.1 to 0.4)	0.0 (−0.1 to 0.0)	—
M2	−0.3 (−0.3 to −0.2)	−0.6 (−0.7 to −0.5)	−0.9 (−1.0 to −0.8)	0.0 (0.0 to 0.1)	0.0 (−0.2 to 0.2)	0.2 (−0.1 to 0.4)	0.0 (−0.1 to 0.0)	0.0^d^ (0.0 to 0.0)
Mean mood logs								
M1	−1.5^d^ (−1.7 to −1.2)	−2.8^d^ (−3.1 to −2.6)	−4.3^d^ (−4.6 to −4.1)	0.2 (0.0 to 0.4)	1.9^d^ (1.0 to 2.8)	1.6^d^ (0.6 to 2.6)	−0.1 (−0.3 to 0.0)	—
M2	−1.5^d^ (−1.7 to −1.2)	−2.8^d^ (−3.1 to −2.6)	−4.3^d^ (−4.6 to −4.1)	0.2 (0.0 to 0.4)	1.9^d^ (1.0 to 2.8)	1.6^d^ (0.6 to 2.6)	−0.1 (−0.3 to 0.0)	0.0 (0.0 to 0.0)
Mean meal logs								
M1	−0.6^d^ (−0.7 to −0.5)	−1.0^d^ (−1.1, −0.9)	−1.6^d^ (−1.7 to −1.5)	0.1^d^ (0.0 to 0.2)	0.6^d^ (0.3 to 0.9)	0.6^d^ (0.3 to 0.9)	0.0 (−0.1 to 0.0)	—
M2	−0.6^d^ (−0.7 to −0.5)	−1.0^d^ (−1.1 to −0.9)	−1.6^d^ (−1.7 to −1.5)	0.1^d^ (0.0 to 0.2)	0.6^d^ (0.3 to 0.9)	0.6^d^ (0.3 to 0.9)	0.0 (−0.1 to 0.0)	0.0 (0.0 to 0.0)
Mean phone logs								
M1	−2.5^d^ (−2.9 to −2.1)	−4.6^d^ (−5.0 to −4.2)	−6.7^d^ (−7.1 to −6.3)	0.3 (0.1 to 0.6)	2.8^d^ (1.5 to 4.2)	2.2^d^ (0.7 to 3.8)	−0.2 (−0.5 to 0.0)	—
M2	−2.5^d^ (−2.9 to −2.1)	−4.6^d^ (−5.0 to −4.2)	-6.7^d^ (−7.1 to −6.3)	0.3 (0.1 to 0.6)	2.8^d^ (1.4 to 4.2)	2.2^d^ (0.7 to 3.8)	−0.2 (−0.5 to 0.0)	0.0 (0.0 to 0.1)
Mean watch logs								
M1	—	−0.3^d^ (−0.3 to −0.2)	−0.5^d^ (−0.5 to −0.4)	0.0 (0.0 to 0.1)	−0.1 (−0.2 to 0.1)	−0.1 (−0.3 to 0.1)	0.0 (0.0 to 0.0)	—
M2	—	−0.3^d^ (−0.3 to −0.2)	−0.5^d^ (−0.5 to −0.4)	0.0 (0.0 to 0.1)	−0.1 (−0.2 to 0.1)	−0.1 (−0.3 to 0.0)	0.0 (0.0 to 0.0)	0.0 (0.0 to 0.0)
Mean use								
M1	−2.2^d^ (−2.6 to 1.8)	4.5^d^ (−4.9 to −4.1)	−6.8^d^ (−7.2 to −6.4)	0.3 (0.0 to 0.6)	2.8^d^ (1.4 to 4.2)	2.2^d^ (0.6 to 3.8)	−0.2 (−0.5 to 0.0)	—
M2	−2.2^d^ (−2.6 to 1.8)	4.5^d^ (−4.9 to −4.1)	−6.8^d^ (−7.3 to −6.4)	0.3 (0.0 to 0.6)	2.8^d^ (1.3 to 4.2)	2.2^d^ (0.6 to 3.8)	−0.2 (−0.5 to 0.0)	.03 (−0.02 to 0.08)

a Age and current BMI regression coefficients and standard errors were calculated based on 5-year and 5kg/m2 differences, respectively, to facilitate interpretation for more meaningful differences in engagement.

b M1: model 1 presents the conditional models using demographic variables as predictors.

c M2: model 2 presents conditional models with the demographic variables and baseline binge episodes as predictors.

d Indicates values that are significant.

e Not applicable.

## Discussion

### Principal Findings

This study described various measures of engagement with a digital ED app, recovery record, and deepened our understanding of how individuals with binge eating use different components and delivery methods of the app. All measures of engagement declined over the course of the study, consistent with trends observed in other digital ED and mental health interventions; however, participants engaged with the app for an average of 3.5 weeks, which was greater than expected given that most digital mental health interventions observe engagement over fewer days [40-42]. Several baseline variables emerged as significant predictors of unique measures of engagement, highlighting the importance of more nuanced assessments of engagement with digital ED interventions. Findings for each aim are discussed in turn below, followed by notable discussion points for measures of engagement and study limitations.

Although every measure of engagement declined, the percentage of participants in this study that were still using recovery record at the end of 4 weeks (209/893, 23%) was substantially greater than that observed in other self-monitoring apps (6%) [40]. Across log types, mean behavior and urge logs had the largest percent reduction over 4 weeks, which could reflect both the overall decline in engagement in combination with decreased ED symptomatology as observed in the BEGIN feasibility study [29]. In addition, participants logged an average of 3 meals/day, and mean meal logs had the smallest percent reduction (1-[2.35/3.58], 34%) of any measure of engagement over the course of 4 weeks, illustrating that most participants were on track with a regular eating treatment target in cognitive behavioral ED treatments [33].

Mood logs were the most frequently used logs, accounting for 62% of all logs, which is in part explained by participants having the ability to create a unique mood log for each emotion as part of meal or behavior log, or as a separate mood log altogether. The greater number of opportunities to log moods in addition to the more limited nature of when meals and ED behaviors or urges occur may in large part explain the vast difference in sample sizes between log types. When measuring engagement across device type, participants completed substantially more phone logs than watch logs, which could partially be explained by meal logs only being offered on the phone. However, the percent reduction in watch logs was 35% greater than the percent reduction of phone logs over the last 3 weeks, demonstrating that the decline in watch engagement was much steeper than the phone engagement. Although the watch app was designed to improve discreetness of completing logs while simultaneously enhancing the usability of and engagement with the app, qualitative research may be necessary to understand what maintained participants’ engagement on their phones more than on the watch.

Second, the engagement trajectory visualizations illustrated that most of the engagement measures had similar overall downward trajectories. In conjunction with weekly summary data presented in Table 2, the spaghetti plots exposed the individual variability in engagement trajectories and underscored that not all components of the app were used the same over the course of a month. Although each measure of engagement used a linear functional form for subsequent multilevel models, the most variation in individual trajectories was observed in mean meal logs, which could vary tremendously on an individual’s availability to complete a more time-intensive log. As an extension of this aim in future work, engagement visualizations may serve useful in characterizing subgroups of engagement profiles through a repeated measures latent profile analysis and can subsequently be used to identify unique engagement and symptom profiles associated with positive intervention outcomes (Peiper et al [43] for an example in treatment-resistant depression). Subsequent studies may also seek to visualize digital intervention usage at more granular levels in terms of time and within individuals, which would be essential to tailor interventions to individual users and to provide just-in-time adaptive interventions that are responsive to engagement.

Third, the multilevel models yielded results that primarily illustrated that time was the best predictor of engagement. Time, measured in weeks, was a significant predictor in every model, and the addition of other predictors to the models typically did not improve model fit. This result was unsurprising because a consistent theme in digital intervention literature is that engagement drops as time progresses [40,44]. Beyond time, sex and ethnicity were the most common significant baseline predictors of engagement. Those who were assigned as female based on genotyping or self-identified as Hispanic were more engaged than their male and non-Hispanic counterparts, respectively, for 4 measures of engagement: mean meal logs, mean mood logs, mean phone logs, and mean use. Although there is little research evaluating baseline predictors of engagement in digital ED interventions, 1 recent study found that sex and ethnicity did not predict engagement when defined as a dichotomous variable of greater or less than 10 minutes of app usage [45]. However, there is some evidence to suggest sex and ethnicity are significant predictors of engagement in other digital mental health interventions [eg, 46,47]. A key point to consider is how the design of the app may have biased engagement toward demographic subgroups (eg, cultural adaptations may increase engagement of racial or ethnic groups). Even though acceptance-facilitating interventions can increase participants’ acceptance, motivations, and positive attitudes toward digital ED interventions regardless of demographic characteristics, initial work in this area highlights that engagement does not improve [48], underscoring the importance of using user-centered design principles for improving engagement for target demographics [49].

A second set of baseline characteristics included significant predictors of only 1 measure of engagement each. Current BMI was a significant negative predictor of mean urge logs, and this result could be interpreted as those with higher BMIs may not have experienced as many urges or did not log their urges as often as those with lower BMIs. Similarly, the number of baseline binge episodes was a significant predictor of the mean behaviors logged, possibly reflecting that those who had greater mean behavior logs over time were more engaged in the app. However, an alternative explanation is that participants with a higher number of baseline binge episodes had more opportunities to log behaviors. To disentangle these results in future studies, it will be essential to assess and compare retrospective self-reported urges and behaviors over a given interval to the behaviors logged in the moment via digital intervention. Notably, the addition of binge episodes at baseline to the second set of engagement models did not improve fit statistics, illustrating little to no contribution to improvement in engagement prediction.

Finally, age was only a significant predictor of mean meal logs, with older individuals logging more meals than younger counterparts, which was somewhat surprising given the technological literacy of younger generations. Although age, current BMI, and binge episodes at baseline were only significant predictors in 1 engagement model each, these results support findings from machine learning algorithms recently identifying age and measures of ED symptom severity as significant predictors of engagement with and drop out from another digital ED intervention [50]. An important caveat to these 3 results is that the parameter estimates for age, current BMI, and number of binge episodes at baseline hovered around 0, warranting future research as to their clinical and practical use.

Three additional points are worth noting. First, participants used the phone to engage with Recovery Record far more than the watch. Despite being confounded by the recruitment of previous recovery record users who only had access to the phone app, the percent change and more consistent engagement with the phone illustrate that participants were more inclined to use this method of delivery. It is possible that participants may have been less used to wearing or using wearable technology, thus turning to the iPhone version of the app more often. Relatedly, more app content (ie, meal logs) was available through the iPhone app, which also may have contributed to the engagement patterns observed across devices. The models of mean phone and watch logs also demonstrated that 2 baseline characteristics (sex and ethnicity) may be used to help differentiate subsequent engagement depending on the delivery platform, suggesting that design is important for delivering ED interventions through wearables and smartphones. Second, meal logs were unique compared with the other measures of engagement for a few reasons: this measure had the smallest percent reduction over 4 weeks, demonstrated the most variability of participant trajectories as evidenced by the spaghetti plots, and was significantly predicted by 3 baseline demographic predictors (age, sex, and ethnicity). Although this study is an exploratory investigation into expanding how we define engagement, this collection of findings warrants future replication and qualitative studies in other samples with ED psychopathology to evaluate if and why meal logs are consistently and proportionally more used than other components. Finally, an important point about mean use is that several multilevel models had the same significant baseline predictors, suggesting that overall engagement results may be reflecting groups of individuals with similar characteristics engaging with specific digital intervention functions in similar capacities. This may also mean that using overall measures of engagement obscures other results that are more sensitive or have less power. Taken together, our findings underscore that engagement with a digital ED intervention is more nuanced and complex than this research often presents when describing engagement through a single measure and is worth deeper exploration to optimize what individuals gain from using a digital ED intervention.

### Limitations

Overall engagement with various functions of the app was high, particularly the number of days used. A key limitation that could partially explain this observation is that many participants who entered the study had already used the app, and they were required to complete 3 logs in Recovery Record before enrolling in the study. Therefore, the baseline level of engagement may be higher than what would be observed outside of a research study. In addition, participants’ greater likelihood of engaging could be due to the convenience and accessibility of using the app on both the phone and the watch coupled with the relative ease of completing simple functions compared with other digital eating disorder interventions (ie, logging a behavior is easier and quicker than completing a guided self-help session in an app). Future studies may consider comparing the same granularity of engagement with new and existing users of digital interventions. In addition, the measures of engagement had significant overlap in the data used to test the multilevel models, so it was unsurprising to observe patterns across the significance of baseline predictors. To address this in future work, it may be useful to conduct split-half studies where the discovery sample would identify significant predictors and these models would be tested with the replication sample. Finally, another limitation is that the 4 measures of engagement where sex and ethnicity were significant predictors of engagement had the largest sample size of logs, which could indicate that these models were overpowered. An alternative explanation is that enough data were acquired to consistently detect sex and ethnicity as significant predictors, and the amount of data collected in the other 3 models was insufficient or lacked sufficient variability to detect differences. Future studies may seek to replicate the current findings before evaluating thresholds to determine what baseline characteristics are clinically useful in identifying meaningful changes in engagement. Finally, the findings of this study should be considered in the context that the sample was primarily White, non-Hispanic women with binge-type EDs, so generalizability may be limited to broader demographics and other EDs. Relatedly, engagement associated with gender was not evaluated in this study; however, understanding the engagement patterns of gender-diverse populations will be critical to tailoring content that reflects their experiences and facilitating acceptance of the intervention [51].

### Conclusion

This study provided a novel view of engagement that characterized participants’ usage of different functions, method of delivery, and overall usage of a digital ED intervention. Key predictors, time, and 2 demographic predictors (sex and ethnicity) were consistently significant despite unique measurements of engagement across log type, device type, and overall engagement. Other baseline demographic and severity of illness predictors were significant in only 1 measure of engagement each, highlighting opportunities to tease out more complex and nuanced understanding of the use of and engagement with different functions of a digital ED app. Future work may consider mixed qualitative and quantitative approaches to better understand and enhance engagement. In addition, identifying unique engagement profiles that, in combination with baseline characteristics, can be used to predict intervention outcomes may allow researchers and clinicians to intervene earlier on engagement and harmful eating behaviors. Considering the importance of consistent engagement in traditional psychotherapy, which is required for meaningful symptom change, this study sets the stage for understanding what types of engagement with digital ED interventions may be most helpful and can reliably predict change in symptoms.

## Supplementary material

10.2196/68824 Multimedia Appendix 1 Spaghetti plots of each engagement measure across 4 weeks.

10.2196/68824 Multimedia Appendix 2 Model fit statistics for baseline predictors of engagement.

## Abbreviations

AIC: Akaike Information Criterion
AN: anorexia nervosa
BED: binge-eating disorder
BEGIN: Binge Eating Genetics Initiative
BIC: Bayesian Information Criterion
BN: bulimia nervosa
ED: eating disorder
EDE-Q: Eating Disorder Examination Questionnaire v6
FDR: False Discovery Rate
ICC: intraclass correlation
