# Supplementary material for: How Engagement Changes Over Time in a Digital Eating Disorder App: Observational Study
Source: JMIR Mhealth Uhealth. 2025 Sep 30;13:e68824. doi: 10.2196/68824 (PMC12483339; doi:10.2196/68824)
Supplement: Multimedia Appendix 1 [file mhealth-v13-e68824-s001.docx]

**Figure S1: Spaghetti plots of each engagement measure across four weeks.**


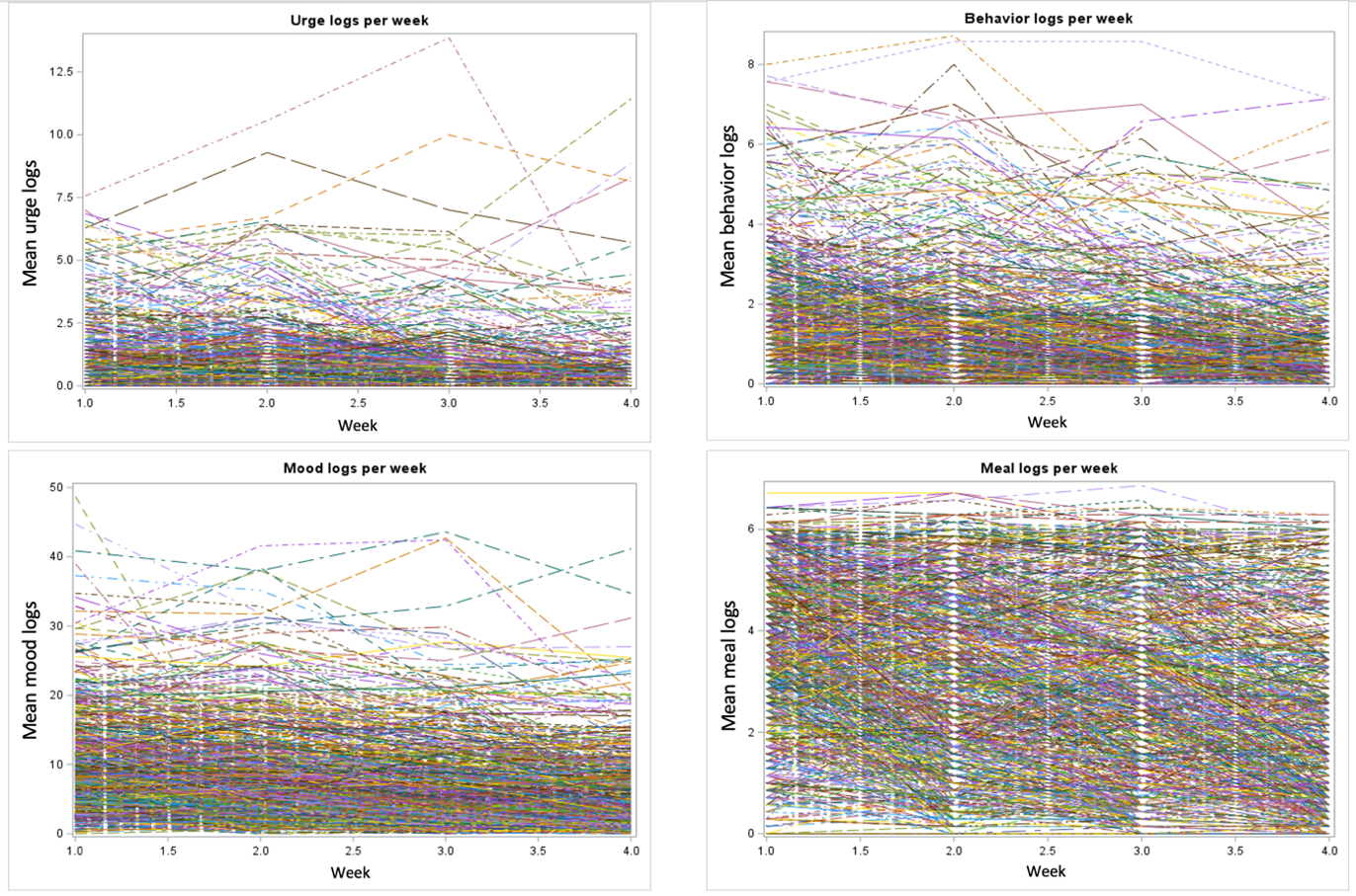

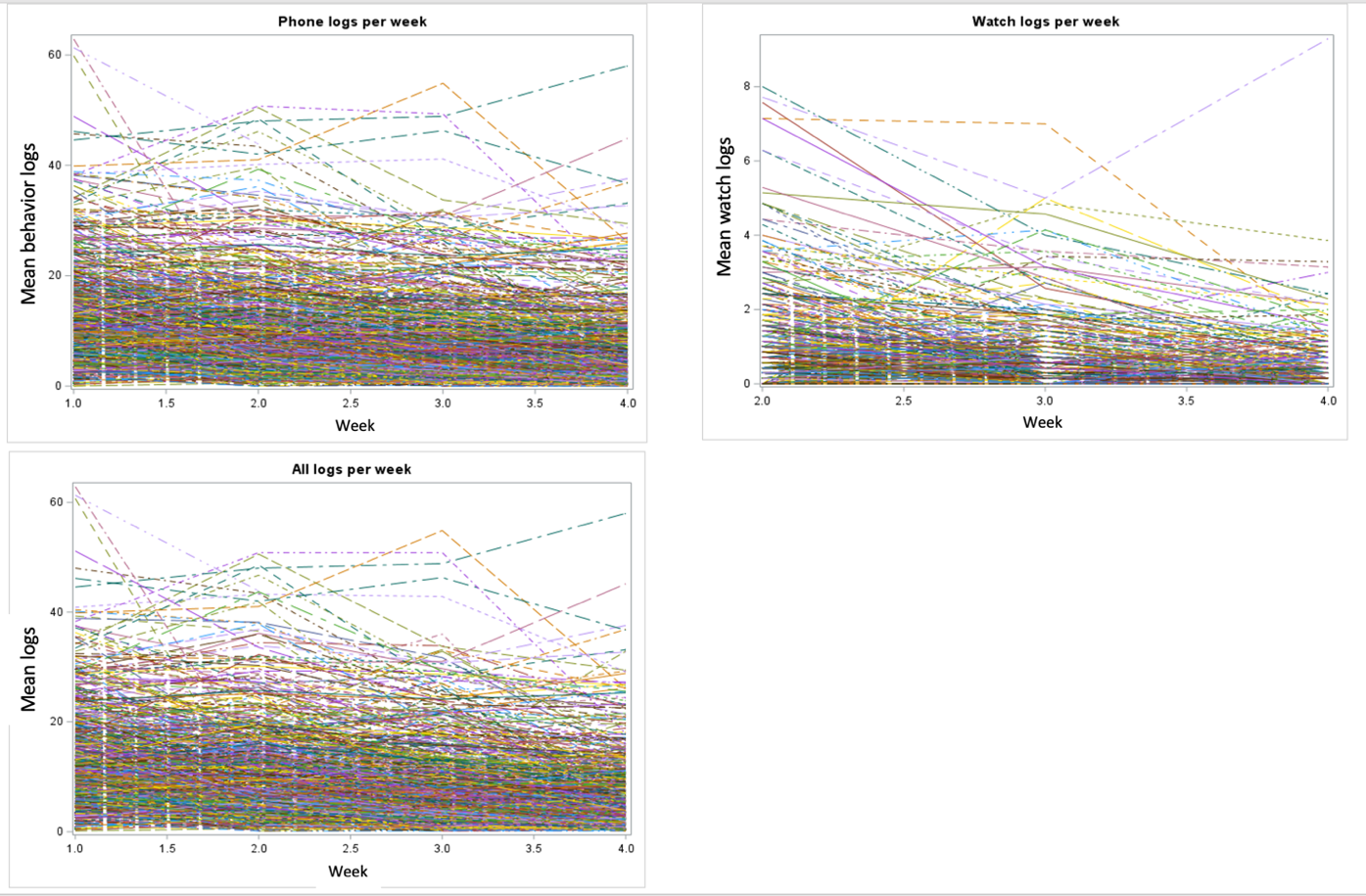


Note: the watch log spaghetti plot only uses data aggregated from weeks 2, 3, and 4 since participants received their Apple Watch devices by the end of week 1.
