# Supplementary material for: How Engagement Changes Over Time in a Digital Eating Disorder App: Observational Study
Source: JMIR Mhealth Uhealth. 2025 Sep 30;13:e68824. doi: 10.2196/68824 (PMC12483339; doi:10.2196/68824)
Supplement: Multimedia Appendix 2 [file mhealth-v13-e68824-s002.docx]

**Supplementary Table 1: Model fit statistics for baseline predictors of engagement.**

| **Engagement Variable** | **Model** | **ICC** | **AIC** | **BIC** |
| --- | --- | --- | --- | --- |
| Mean urge logs | Null | 0.58 | 8545.3 | 8554.9 |
|  | Model 1 | 0.61 | 8356.3 | 8365.9 |
|  | Model 2 | 0.61 | 8364.5 | 8374.1 |
| Mean behavior logs | Null | .58 | 9416.4 | 9426.0 |
|  | Model 1 | .66 | 8923.0 | 8932.6 |
|  | Model 2 | .65 | 8937.3 | 8946.9 |
| Mean mood logs | Null | .63 | 19441.0 | 19450.6 |
|  | Model 1 | .73 | 18604.7 | 18614.3 |
|  | Model 2 | .73 | 18610.2 | 18619.8 |
| Mean meal logs | Null | .57 | 11654.1 | 11663.7 |
|  | Model 1 | .70 | 10790.7 | 10800.3 |
|  | Model 2 | .70 | 10795.4 | 10805.0 |
| Mean phone logs | Null | .61 | 21068.0 | 21077.6 |
|  | Model 1 | .72 | 20205.6 | 20215.2 |
|  | Model 2 | .72 | 20209.9 | 20219.4 |
| Mean watch logs | Null | .48 | 5412.4 | 5421.8 |
|  | Model 1 | .55 | 5209.8 | 5219.3 |
|  | Model 2 | .55 | 5217.0 | 5226.4 |
| Mean use | Null | .61 | 21246.8 | 21256.4 |
|  | Model 1 | .72 | 20392.7 | 20402.3 |
|  | Model 2 | .72 | 20392.7 | 20402.3 |

AIC: Akaike Information Criterion

BIC: Bayesian information criterion

ICC: Intraclass correlation

Note: Model 1 presents the conditional models using demographic variables as predictors; Model 2 presents conditional models with the demographic variables and baseline binge episodes as predictors.
